# Squamous Cell Carcinoma in the Diabetic Foot: A Review of Published Case Reports

**DOI:** 10.1002/jfa2.70217

**Published:** 2026-10-26

**Authors:** Matthew R. Carroll, Ivan R. Bristow

**Affiliations:** ^1^ Department of Podiatry, School of Allied Health Auckland University of Technology Auckland New Zealand; ^2^ Health Sciences University Bournemouth UK

**Keywords:** diabetic foot, marjolin ulcer, squamous cell carcinoma, verrucous carcinoma

## Abstract

**Introduction:**

Squamous cell carcinoma (SCC) is a malignant tumour arising from epidermal keratinocytes and is the second most common form of skin cancer globally. Although relatively rare on the foot, SCC and its variants may occur in people with diabetes and can mimic common diabetes‐related foot ulcerations. This diagnostic overlap presents a risk of delayed recognition and treatment. The aim of this review was to synthesise published case reports of SCC occurring in the diabetic foot and to describe patient characteristics, clinical presentation, and outcomes.

**Methods:**

A review of case reports was conducted in accordance with the PRISMA guidelines. Electronic databases (Medline, CINAHL, SPORTDiscus and SCOPUS) were searched up to September 2025 using relevant terms for SCC and its variants. Studies describing clinical cases of SCC arising on the foot in individuals with diabetes were included.

**Results:**

Thirty‐one cases from 13 countries were included. The mean age of patients was 62 years, with a predominance of males (71.0%) and type 2 diabetes (64.5%). Lesions most affected the right foot (64.5%) and were frequently located on the toes or plantar surfaces. The mean lesion duration prior to diagnosis was 61.9 months (range 2–360; *n* = 25). Initial misdiagnosis was most commonly as a foot ulcer (54.8%), followed by a wart (19.4%) and osteomyelitis (12.9%). Final diagnoses included SCC (51.7%), verrucous carcinoma (35.5%), and Marjolin's ulcer (12.9%). Pain was reported in 58.1% of cases despite neuropathy being present in 45.2%. Recurrence occurred in 16.1% of cases.

**Conclusions:**

Squamous cell carcinoma in people with diabetes‐related foot disease is rare but frequently misdiagnosed, resulting in prolonged lesion duration prior to diagnosis. Clinicians should maintain a high index of suspicion for malignancy in atypical or non‐healing foot lesions, particularly those with unusual features or persistence despite treatment. Early biopsy is essential for timely diagnosis and optimising outcomes.

## 1 Introduction

Squamous cell carcinoma (SCC) is a malignant tumour originating from epidermal keratinocytes or the skin appendages. Globally, SCC is the second most common skin cancer after basal cell carcinoma (BCC) [1]. Squamous cell carcinoma represents a group of heterogenous diseases in its presentation and cause. Risk factors for its development include advancing age, ultraviolet (UV) radiation, genetic predisposition, physical and chemical carcinogens, immunosuppression, drugs, viral infection, chronic inflammation, and chronic injury of the skin [2]. Although the disease affects all skin types, sun‐exposed areas are the most common sites of development in fair‐skinned individuals.

Squamous cell carcinoma of the foot is relatively uncommon. A 9‐year review of skin tumours reported that SCC accounted for around 8% of all malignant tumours arising on the foot, after melanoma (melanoma representing 83% of cases). Just over 75% of SCCs in the study were located on the soles. Given the limited exposure to UV radiation at these sites, alternative pathogenic mechanisms such as chemical carcinogens, ulceration, injury, viral infection, and chronic inflammation are more likely contributors [3]. Although rare, SCC in the foot carries important clinical implications, as delayed diagnosis may result in significant local tissue destruction, a greater likelihood of extensive surgical intervention, including amputation, and, in some cases, metastatic spread.

Several recognised variants of SCC may arise in the foot. Verrucous carcinoma (Carcinoma cuniculatum) is slow‐growing, well‐differentiated, and rarely metastasises. The tumour may take on an exophytic verrucous form or as a hyperkeratotic or ulcerated lesion. The aetiology is unclear, but studies have shown frequent concurrent Human papillomavirus (HPV) infection (subtypes 11 & 16), which may occur due to chronic inflammation and ulceration [4]. Marjolin's ulcer, in contrast, is an aggressive and regressive neoplasm that typically develops in an area of chronic ulceration, scar tissue, or thermal burn and most commonly presents as SCC [5], although other types of malignancies, such as melanoma, sarcoma and BCC, may occur [6].

Diabetes is a common cause of chronic and recurrent foot ulceration due to microvascular and macrovascular complications [7], leading to neuropathy and ischaemia, or a combination of both. The lifetime risk of developing a diabetic foot ulcer (DFU) is estimated to be between 19% and 34%, with millions of cases occurring globally each year [8]. In this context, distinguishing between benign ulceration and malignant changes presents a significant clinical challenge. Lesions such as SCC may closely mimic DFU in their appearance, including ulceration, hyperkeratosis, and chronic non‐healing behaviour. Given the high prevalence of DFU, clinicians may understandably attribute atypical lesions to common diabetes‐related pathology. Consequently, rare malignancies such as SCC may initially be managed as DFU, potentially delaying definitive diagnosis and treatment.

The relationship between diabetes and skin cancer has been increasingly explored [9] and a growing number of papers highlight skin malignancies mimicking diabetes‐related foot ulcers (DFU) [10]. Proposed mechanisms linking diabetes with malignancy include chronic low‐grade inflammation, impaired immune surveillance, and metabolic dysregulation associated with hyperglycaemia, all of which may contribute to carcinogenesis or delay the recognition of malignant change. Despite this, the extent to which diabetes influences the development, presentation, or outcomes of SCC in the foot remains unclear [10].

Although reports of SCC arising in the diabetic foot are increasing, the existing literature is largely limited to isolated case reports. This fragmented evidence base limits the ability to identify consistent clinical features, risk factors, and diagnostic patterns that could help clinicians distinguish malignant lesions from more common diabetes‐related foot conditions.

Therefore, the aim of this study was to systematically review published case reports of SCC and its variants occurring in the feet of people with diabetes, to synthesise patient characteristics, clinical presentation, and outcomes, and to support improved recognition of malignancy in clinical practice.

## 2 Materials & Methods

### 2.1 Study Design and Reporting

This review was conducted in accordance with the Preferred Reporting Items for Systematic Reviews and Meta‐Analyses (PRISMA 2020) guidelines. The PRISMA checklist is provided in Supporting Information S1. A review protocol was developed a priori but was not formally registered.

### 2.2 Eligibility Criteria

We included English‐language case reports describing individuals with diabetes mellitus presenting with histologically confirmed squamous cell carcinoma (SCC) or its variants (including verrucous carcinoma and Marjolin's ulcer) on the foot distal to the ankle. Case reports were selected to enable detailed extraction of individual patient‐level clinical characteristics, which are often not available in aggregated study designs. Multi‐case reports were eligible where individual case‐level data could be extracted. Studies reporting aggregate data only were excluded. We excluded cohort studies, cross‐sectional studies, conference abstracts, reviews, theses, editorials, letters, and other non‐primary reports.

### 2.3 Search Strategy

A comprehensive search of electronic databases (MEDLINE, CINAHL, SPORTDiscus and Scopus) was conducted from database inception to September 2025. No restrictions were placed on publication date, and the earliest eligible case report identified was published in 1997. The search strategy combined controlled vocabulary terms (e.g., MeSH) and keywords related to ‘squamous cell carcinoma’, ‘verrucous carcinoma’, ‘Marjolin ulcer’, ‘diabetic foot’, and ‘foot ulcer’, adapted for each database. No date restrictions were applied. In addition to database searching, backward citation searching was undertaken by screening the reference lists of all included studies to identify additional eligible case reports. Grey literature was not systematically searched. The full search strategies for all databases are provided in Supporting Information S2.

### 2.4 Study Selection

All records were imported into a reference management system, and duplicates were removed. Two reviewers (I.B. and M.C.) independently screened titles and abstracts for eligibility, followed by full‐text review of potentially relevant articles. Disagreements were resolved through discussion, and consensus was reached among the reviewers. The study selection process is summarised in the PRISMA flow diagram (Figure 1).

**FIGURE 1:**
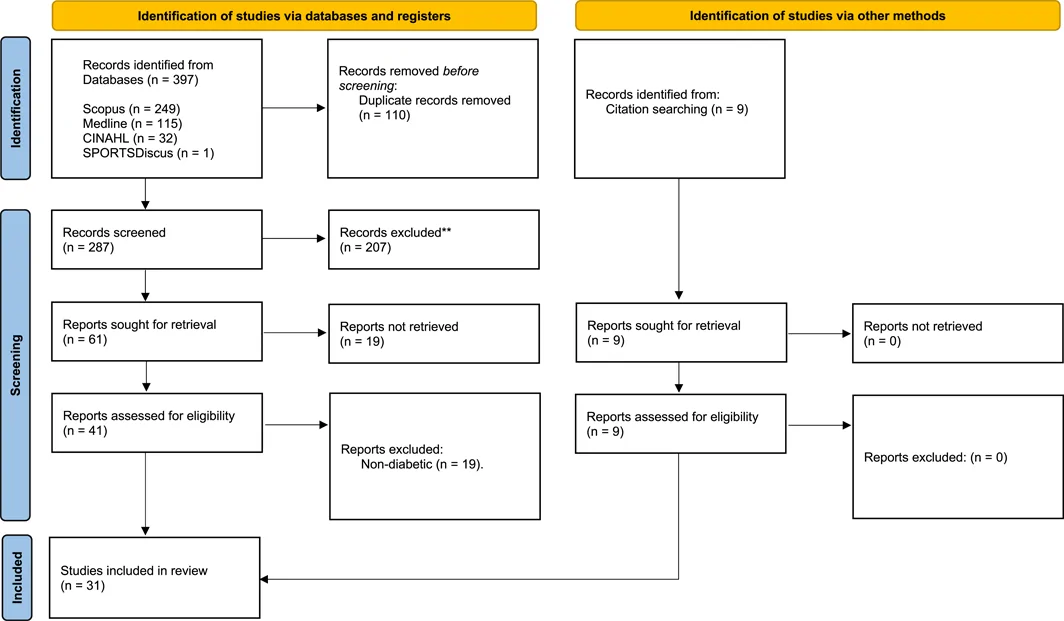
PRISMA flow diagram.

### 2.5 Data Extraction

Extracted data included study characteristics, patient demographics, lesion characteristics, clinical presentation, diagnostic features, treatment, and outcomes (Table 1). Where information was not reported in the original publication, it was recorded as missing, and no attempts were made to impute data.

**TABLE 1:** Study characteristics, demographics, lesion characteristics, and clinical information that were extracted from all included case reports.

Category	Data extracted	Definition
Study characteristics	Author	Name of author
	Year	Year of publication
Demographics	Patient age	Age in years
	Type of diabetes	Type of diabetes
	Ethnicity	Ethnicity of participants
Lesion characteristics & location	Unusual features	Bleeding or hypergranulation tissue, ulcer located in an atypical area
	Location	A lesion that is static or deteriorating despite treatment
	Colour	Irregular colour within, or patchy pigmentation around the ulcer
	Evolution	Enlargement despite treatment
	Risk factors	Risk factors reported
	Malodour or discharge	Yes/no
	Description of lesion	Specific description of the lesion
Clinical information	History of pain	Yes/no
	History of neuropathy/neuropathic ulceration	Yes/no
	History of smoking	Yes/no
	History of trauma	Yes/no
	Imaging features	Specific imaging features reported
	Biopsy reported	Yes/no
	Histological features	Specific histological features reported
	Initial diagnosis	Yes/no
	Initial misdiagnosis	Reporting of misdiagnosis
	Treatment history	Details of treatment provided
	Recurrence	Reported recurrence
	Health status	Alive or deceased

### 2.6 Quality Assessment

The methodological quality of the included case reports was appraised using the Joanna Briggs Institute (JBI) Critical Appraisal Checklist for Case Reports [11]. This tool evaluates key domains, including patient demographics, clinical history, diagnostic methods, interventions, and outcomes. All included case reports were initially independently reviewed by both authors. Following this, a consensus meeting was held to discuss each case in detail and to agree on the interpretation of the reported clinical information and key methodological features. Once consensus was reached, a formal quality appraisal was undertaken. Data extraction support was assisted by Microsoft Copilot (Microsoft Corporation). Copilot was used to facilitate structured information extraction and to assist in populating the Joanna Briggs Institute (JBI) critical appraisal checklist for each included case report. AI‐assisted outputs were reviewed, verified, and amended where necessary by the reviewer to ensure accuracy and consistency with the source publications. Final appraisal decisions and scoring were made exclusively by the authors.

### 2.7 Data Synthesis

Given the heterogeneity of case reports and variability in reporting, a narrative synthesis was undertaken. Descriptive statistics, including frequencies, percentages, and means, were used to summarise patient characteristics, lesion features, and clinical outcomes. Where data were incomplete, analyses were conducted using available case numbers for each variable.

### 2.8 Consideration of Bias

As this review was based on published case reports, findings are subject to potential publication and reporting bias. Cases with unusual presentation or outcomes may be more likely to be reported, which may limit generalisability.

## 3 Results

In this review, we identified 397 records using the predetermined search terms. Figure 1 elaborates the screening and selection process in the PRISMA flow diagram. Table 2 summarises the patient demographics and characteristics. The 31 published cases [12, 13, 14, 15, 16, 17, 18, 19, 20, 21, 22, 23, 24, 25, 26, 27, 28, 29, 30, 31, 32, 33, 34, 35, 36, 37, 38, 39, 40, 41] represented data from 13 countries, with 47% (*n* = 15) of cases reported from the United Kingdom and the United States. The average age of patients was 62 years, with 20 (64.5%) having type 2 diabetes. Ethnicity was reported in only 25.8% (*n* = 8) of the included cases. Of these, four patients were reported as Black/African American, three as White/Caucasian, and one as Asian; ethnicity was not reported for 74.2% (*n* = 23) of cases.

**TABLE 2:** Patient demographics and diabetes characteristics (*n* = 31).

Variable	Category	*n* (%)
Sex	Male	22 (71.0)
	Female	9 (29.0)
Age (years, mean)		62
Ethnicity	Black/African American	4 (12.9)
	White/Caucasian	3 (9.6)
	Asian	1 (3.2)
	Not reported	23 (74.1)
Diabetes type	Type 1	6 (19.4)
	Type 2	20 (64.5)
	Not reported	5 (16.1)
Country of report	UK	5 (16.1)
	USA	10 (32.3)
	Other countries	16 (53.0)

Table 3 displays the lesion characteristics and location. The most prevalent locations on the foot were the toes (*n* = 6, 19.4%) and the hallux (*n* = 5, 16.1%). For lesions with reported longevity, the mean duration was 61.92 months (range 2–360 months; *n* = 25).

**TABLE 3:** Lesion characteristics and location.

Variable	Category	*n* (%)
Laterality	Left foot	10 (32.3)
	Right foot	20 (64.5)
	Bilateral	1 (3.2)
Anatomical location	Toes	6 (19.4)
	Hallux	5 (16.1)
	Plantar rearfoot	5 (16.1)
	Plantar forefoot	4 (12.9)
	Nail unit	3 (9.7)
	Dorsal surface	1 (3.2)
	Amputation stump	1 (3.2)
Lesion duration (months)^a^	Mean (range)	61.92 (2–360)

^a^ Reported in *n* = 25 case reports.

Table 4 summarises the clinical characteristics and initial diagnoses of the lesions. Peripheral neuropathy or neuropathic ulceration was reported in 17 cases (54.8%). Despite the presence of neuropathy, pain was documented in 18 cases overall (58.1%), including seven (41.2%) of those with neuropathy or neuropathic ulceration. Before the correct diagnosis was established, lesions were most commonly misdiagnosed as diabetic foot ulcers (DFUs) (*n* = 17, 54.8%). Malodour was also frequently reported, occurring in 11 cases (35.5%).

**TABLE 4:** Clinical presentation and initial misdiagnosis.

Variable	Category	*n* (%)
Neuropathy or neuropathic ulceration	Present	17 (54.8)
	Not reported/absent	14 (45.2)
Pain	Present	18 (58.1)
	Absent	5 (16.1)
	Not reported	8 (25.8)
	Present in cases with neuropathy	7 (41.1)
Malodour	Present	11 (35.5)
	Not reported/absent	20 (64.5)
Initial misdiagnosis	Diabetic foot ulcer	17 (54.8)
	Wart	6 (19.4)
	Osteomyelitis	4 (12.9)
	Other/not stated	4 (12.9)

Table 5 presents the final diagnoses and outcomes. Overall, 51.7% (*n* = 16) were confirmed as SCC, 35.5% (*n* = 11) as verrucous carcinoma, and 12.9% (*n* = 4) as a Marjolin's ulcer. Recurrence after treatment occurred in five patients (16.1%). Five patients (16.1%) were reported as alive at the conclusion of the report, four as deceased (12.9%), and 22 cases (71%) had an unreported status.

**TABLE 5:** Final diagnosis and outcomes.

Variable	Category	*n* (%)
Final diagnosis	Squamous cell carcinoma (SCC)	16 (51.7)
	Verrucous carcinoma	11 (35.5)
	Marjolin's ulcer	4 (12.9)
Recurrence	Yes	5 (16.1)
	No	15 (48.4)
	Not reported	11 (35.5)
Survival status	Alive	5 (16.1)
	Deceased	4 (12.9)
	Not reported	22 (71.0)

### 3.1 Methodological Quality of Included Studies

The methodological quality of the included case reports was generally high. Using the JBI Critical Appraisal Checklist for Case Reports, the mean quality score was 7.2/8 (median 7/8; range 5–8). Thirteen studies (42%) were rated as high quality (8/8), 11 (35%) as good quality (7/8), and seven (23%) as moderate quality (≤ 6/8), with no studies classified as low quality. Most reports provided detailed descriptions of patient demographics, clinical presentation, diagnostic assessment, and treatment, including histopathological confirmation. However, reporting of adverse events and long‐term follow‐up was inconsistent. Full appraisal results are provided in Supporting Information S3.

## 4 Discussion

This study provides a review of 31 case reports on SCC and its variants arising on the feet of people with diabetes. The mean age of patients at diagnosis was 62.7 years, which broadly aligns with previous research reporting that SCC is most common in the 5th and 6th decades of life [42, 43, 44]. A predominance of male patients was also observed, consistent with evidence that SCC occurs approximately three times more frequently in males than females [45].

Ethnicity was reported in only 25.8% (*n* = 8) of cases, with four patients identified as Black/African American, three as White/Caucasian, and one as Asian. No case report formally classified patients according to a recognised dermatological skin‐type system such as the Fitzpatrick phototype scale. Consequently, it was not possible to explore whether skin type, ethnicity, socioeconomic status, or disparities in access to diabetic foot services influenced delays in diagnosis or treatment. Given the recognised inequities in access to specialist diabetic foot care globally, more comprehensive reporting of demographic and socioeconomic characteristics in future case reports would assist in understanding their contribution to disease presentation and outcomes.

The review found that the most frequent misdiagnosis was DFU (54.8%). This finding reinforces the central observation of this review: SCC and its variants may closely mimic common diabetes‐related foot pathology. Given the substantially higher prevalence of DFU compared with SCC, clinicians are understandably more likely to attribute atypical foot lesions to diabetes‐related ulceration rather than malignancy [10]. Similar diagnostic challenges have been reported for melanoma of the foot, where 85% of cases were initially misdiagnosed as DFU [46]. Consequently, SCC arising in the feet of people with diabetes may remain unrecognised for prolonged periods while standard ulcer management strategies are pursued [10].

Diabetes‐related foot disease is a well‐documented condition driven by ischaemia, neuropathy and infection. The consequences of which are often trauma leading to DFU [47]. The lifetime risk of developing a foot ulcer is between 19% and 34% and it is suggested that 9.1 million to 26.1 million individuals with diabetes globally develop foot ulcers every year [8].

The average duration of lesions before diagnosis in this review was 61 months (approximately 5 years), highlighting the substantial delays that can occur before malignancy is recognised. While malignant transformation of chronic wounds is a recognised phenomenon and was observed in four cases classified as Marjolin ulcers [48, 49], this represented only a small proportion of the included cases (12.9%). In contrast, most lesions were primary SCC or verrucous carcinoma occurring in people with diabetes that clinically resembled DFU. The prolonged lesion duration observed across the included cases therefore appears to reflect the difficulty in distinguishing rare malignant lesions from far more common diabetes‐related foot disorders. Although malignant transformation has been reported to occur after many years of chronic inflammation [50, 51] the principal challenge identified by this review was the delayed recognition of malignancy masquerading as DFU.

The majority of SCC lesions (*n* = 20, 66%), where location was reported, occurred on weight‐bearing areas of the plantar foot, such as the heel and forefoot, a distribution similar to that observed in DFU [52]. This overlap in anatomical location may contribute to delayed recognition, as lesions arising within these sites are more likely to be attributed to DFU than malignancy. Lesions were also more commonly reported on the right foot (*n* = 20, 64.5%) than on the left foot (*n* = 10, 32.3%), although this may represent a chance finding due to the small number of cases. Lesion location may also support a traumatic aetiology, as SCC in non‐sun‐exposed sites has been reported to arise in areas of compromised skin, including pressure ulcers, scars, inflammatory lesions, and chronic wounds [49]. Prolonged irritation and inflammation from repeated trauma, particularly within areas of mechanical stress, may provide sufficient chronic stimulation to promote malignant change [18]. Various traumatic, immunological, irritation, and toxin‐based mechanisms have been proposed; however, no definitive aetiology has been established [53].

Within the 31 identified case reports, 11 (35.5%) were histologically classified as verrucous carcinoma (epithelioma cuniculatum). This is a slow‐growing, well‐differentiated SCC which rarely metastasises [54]. The tumour is characterised by an exophytic verruciform appearance; however, it may exhibit hyperkeratosis, ulceration, or a malodorous discharge. The aetiology of verrucous carcinoma is unknown, but it can develop in areas of chronic inflammation. Human papillomavirus has been associated with this tumour elsewhere on the body (including genital, oral and subungual cancers), but the association with oncogenic types of plantar VC has been limited to a few case studies (HPV subtypes 16, 18 and 45) [55]. In the case reports reviewed in this study, VC of the foot was frequently misdiagnosed as a wart.

Outcome data for patients with SCC in this review were poorly reported, with four patients reported as deceased, five still alive at the end of the report and 22 not recorded. Review of the reported deaths showed that three were attributable to metastatic SCC, while two were related to significant underlying comorbidities rather than malignancy itself. Although formal analysis was not possible, fatal cases were often characterised by advanced disease at diagnosis following prolonged lesion duration, whereas several low‐grade tumours with similarly long diagnostic delays achieved favourable outcomes after treatment. This suggests that both diagnostic delay and tumour biology may influence prognosis. To the author's knowledge, no research has identified outcomes in SCC of the foot in people with diabetes compared with those without diabetes. However, evidence regarding whether diabetes is a risk factor for SCC development is limited. A study examining squamous cell carcinoma (SCC) of the head or neck indicated that individuals with diabetes exhibited lower overall 5‐year survival rates compared with those without diabetes (61% vs. 78%) [56]. Conversely, another investigation reported no association between diabetes and SCC outcomes [57].

Although substantial advances in diabetes management and multidisciplinary diabetic foot care have occurred over the past three decades, the published case reports did not demonstrate an obvious reduction in diagnostic delay between 1997 and 2019. Across both earlier and later reports, SCC and its variants were repeatedly misdiagnosed as DFU, verrucae, osteomyelitis, or other benign conditions, with delays to definitive diagnosis frequently measured in years rather than months. Similarly, outcomes appeared to be primarily determined by disease stage at diagnosis rather than the era in which treatment occurred. Cases diagnosed while localised generally achieved favourable outcomes following surgical excision, whereas lesions presenting with bone invasion, nodal involvement, or metastatic disease were associated with amputation, recurrence, and mortality. Taken together, these findings suggest that despite major advances in diabetic foot care, the principal challenge remains recognising malignancy within a chronic or atypical foot lesion and undertaking timely biopsy.

This review has emphasised that the principal clinical challenge is recognising SCC and its variants when they masquerade as DFU. Although malignant transformation of chronic wounds was identified in a small number of cases, most reports described primary SCC or verrucous carcinoma that was initially mistaken for DFU or other benign foot pathology. The nature of the tumour means that lesions may be present for months or years before a definitive diagnosis is established. Although their likelihood of metastasis is lower than that of melanoma, these lesions can lead to significant local tissue destruction and are often associated with considerable pain. Comprehensive evaluation of patients presenting with a DFU, especially among older adults, remains essential.

### 4.1 Assessment of Risk Factors

A recent publication [46] describes the application of the acronym ‘U.L.C.E.R’ to raise suspicion for melanoma masquerading as DFU; this approach may also be beneficial for identifying other malignancies, such as squamous cell carcinoma (SCC), which can exhibit similar characteristics. Table 6 provides the definition of the U.L.C.E.R acronym.

**TABLE 6:** The U.L.C.E.R acronym.

U	Unusual	Spontaneous bleeding or hypergranulation tissue, ulcer located in an atypical area for ischaemic or neuropathic lesions
L	Longevity	A lesion that is static or deteriorating despite treatment
C	Colour	Irregular colour within, or patchy pigmentation around the ulcer
E	Evolution	Or enlargement despite treatment
R	Risk factors	For diabetic ulceration, factors such as neuropathy, ischaemia, or infection may be absent

Careful assessment of lesion‐related risk factors is essential in people with diabetes‐related foot disease. In the cases reviewed, neuropathy, a common complication of diabetes, was reported in 14 cases (45.2%). However, pain associated with the lesion was documented in 18 cases (58%), which is atypical in a neuropathic foot where sensation is usually diminished or absent due to sensory nerve damage [58]. The occurrence of pain in these cases may therefore signal the need for further investigation and reinforce the importance of comprehensive clinical assessment.

## 5 Conclusion

This review has evaluated 31 cases of misdiagnosed SCC arising in people with diabetes‐related foot disease. Despite its rarity, SCC can be painful in the diabetic foot and locally destructive, often requiring surgery. As with all malignancies, early recognition is the key to improved outcomes. Careful assessment of all lesions presenting as DFU is required, particularly in older adults, to detect subtle signs of malignancy, including prolonged persistence despite treatment and unusual features. When in doubt, a biopsy is the key to making the diagnosis.

## Author Contributions

**Matthew R. Carroll:** conceptualization, methodology, investigation, writing – review and editing. **Ivan R. Bristow:** conceptualization, methodology, investigation, writing – original draft, writing review and editing.

## Funding

The authors have nothing to report.

## Conflicts of Interest

The authors declare no conflicts of interest.

## Supporting information


Supporting Information S1

Supporting Information S2

Supporting Information S3

## Data Availability

The data that support the findings of this study are available from the corresponding author upon reasonable request.

## References

[1] S. Huang , J. Jiang , H. Wong , P. Zhu , X. Ji , and D. Wang , “Global Burden and Prediction Study of Cutaneous Squamous Cell Carcinoma From 1990 to 2030: A Systematic Analysis and Comparison With China,” Journal of Global Health 14 (2024): 04093, 10.7189/jogh.14.04093. PMC11063968 38695259

[2] A. S. Lonsdorf and E. N. Hadaschil , “Squamous Cell Carcinoma and Keratoacanthoma,” in Fitzpatricks Dermatology, ed. S. Kwang (2019), 1901–1919.

[3] H. K. Park , Y. D. Choi , and S. J. Yun , “Clinical Characteristics and Differences of 802 Acral Tumours, Categorized by Anatomical Sites,” Clinical and Experimental Dermatology 47, no. 2 (2022): 312–318, 10.1111/ced.14885. 34388274

[4] C. E. Griffiths , Rooks's Textbook of Dermatology. 9th ed. (Wiley, 2016).

[5] A. Kowal‐Vern and B. K. Criswell , “Burn Scar Neoplasms: A Literature Review and Statistical Analysis,” Burns 31, no. 4 (2005): 403–413, 10.1016/j.burns.2005.02.015. 15896501

[6] M. Marietta and J. S. Crane , Marjolin Ulcer (2025) [cited 2025 30 November], https://www.ncbi.nlm.nih.gov/books/NBK532861/. 30422456

[7] K. Papatheodorou , M. Banach , E. Bekiari , M. Rizzo , and M. Edmonds , “Complications of Diabetes 2017,” Journal of Diabetes Research 2018, no. 1 (2018): 3086167, 10.1155/2018/3086167. PMC5866895 29713648

[8] D. G. Armstrong , A. J. M. Boulton , and S. A. Bus , “Diabetic Foot Ulcers and Their Recurrence,” New England Journal of Medicine 376, no. 24 (2017): 2367–2375, 10.1056/NEJMra1615439. 28614678

[9] E. C. Dobrică , M. L. Banciu , V. Kipkorir , et al., “Diabetes and Skin Cancers: Risk Factors, Molecular Mechanisms and Impact on Prognosis,” World Journal of Clinical Cases 10, no. 31 (2022): 11214–11225, 10.12998/wjcc.v10.i31.11214. PMC9649529 36387789

[10] A. V. Lyundup , M. V. Balyasin , N. V. Maksimova , et al., “Misdiagnosis of Diabetic Foot Ulcer in Patients With Undiagnosed Skin Malignancies,” International Wound Journal 19, no. 4 (2022): 871–887, 10.1111/iwj.13688. PMC9013580 34713964

[11] E. Aromataris , C. Lockwood , K. Porritt , B. Pilla , and Z. Jordan , eds., JBI Manual for Evidence Synthesis (JBI, 2024), 10.46658/JBIMES-24-01.

[12] F. Ahmedy , K. H. Teoh , N. Azizan , N. Lah , “Foot Squamous Cell Carcinoma: Grieve Lessons From Diabetic Foot Ulcer Misdiagnosis,” Gazi Medical Journal 30, no. 4 (2019).

[13] J. Bentley , “The Remarkable Case of Verrucous Carcinoma—A Rare Find (Poster),” in Wounds UK Conference (2015).

[14] M. Bhushan , J. E. Ferguson , C. E. Hutchinson , and H. L. Muston , “Carcinoma Cuniculatum of the Foot Assessed by Magnetic Resonance Scanning,” Clinical and Experimental Dermatology 26, no. 5 (2001): 419–422, 10.1046/j.1365-2230.2001.00850.x. 11488831

[15] R. Cavaliere , D. M. Mercado , and M. Mani , “Squamous Cell Carcinoma From Marjolin's Ulcer of the Foot in a Diabetic Patient: Case Study,” Journal of Foot and Ankle Surgery 57, no. 4 (2018): 838–843, 10.1053/j.jfas.2017.11.016. 29503134

[16] H. Y. Chiao , S.‐C. Chang , C.‐H. Wang , Y.‐S. Tzeng , and S.‐G. Chen , “Squamous Cell Carcinoma Arising in a Diabetic Foot Ulcer,” Diabetes Research and Clinical Practice 104, no. 2 (2014): e54–e56, 10.1016/j.diabres.2013.12.027. 24630618

[17] Y. S. Chong , S. W. Ahn , M. N. Kim , B. In Ro , and K. Y. Song , “A Case of Verrucous Carcinoma Occurring in Patient With Diabetes Mellitus,” Annals of Dermatology 13, no. 3 (2001): 190–192, 10.5021/ad.2001.13.3.190.

[18] J. W. Christopher , “Diagnostic Dilemmas. Nonhealing Ulcer in a Diabetic Foot,” Wounds: A compendium of clinical research and practice 16, no. 6 (2004): 212–217.

[19] V. Di Palma , J. P. Stone , A. Schell , and J. C. Dawes , “Mistaken Diabetic Ulcers: A Case of Bilateral Foot Verrucous Carcinoma,” Case reports in dermatological medicine 2018 (2018): 1–3: 4192657–4192657, 10.1155/2018/4192657. PMC5896404 29796321

[20] J. Ho , D. G. Diven , P. J. Butler , and S. K. Tyring , “An Ulcerating Verrucous Plaque on the Foot. Verrucous Carcinoma (Epithelioma Cuniculatum),” Archives of Dermatology 136, no. 4 (2000): 547–548, 10.1001/archderm.136.4.547-a. 10768657

[21] R. D. Holgado , S. C. Ward , and S. G. Suryaprasad , “Squamous Cell Carcinoma of the Hallux,” Journal of the American Podiatric Medical Association 90, no. 6 (2000): 309–312, 10.7547/87507315-90-6-309. 10881463

[22] C. Jones and M. Young , “Squamous Cell Carcinoma in a Chronic Diabetic Foot Ulcer,” Diabetic Foot 23, no. 3 (2020): 10–12.

[23] M. F. Kong , R. Jogia , V. Nayyar , R. Berrington , and S. Jackson , “Squamous Cell Carcinoma in a Heel Ulcer in a Patient With Diabetes,” Diabetes Care 31, no. 7 (2008): e57, 10.2337/dc08-0284. PMC2453643 18594056

[24] B. Larijani , S. M. Tavangar , and F. Bandarian , “Squamous Cell Carcinoma Arising in a Chronic, Nonhealing Diabetic Foot Ulcer,” Wounds 29, no. 7 (2017): E48–E50. 28759430

[25] K. MacIntyre , T. Goodfellow , and I. Bristow , “Case Report: Squamous Cell Carcinoma or Diabetic Foot Ulcer?,” Diabetic Foot Journal 11, no. 1 (2008): 22–24.

[26] A. Mantovani , I. Teobaldi , V. Stoico , et al., “Cutaneous Squamous Carcinoma in a Patient With Diabetic Foot: An Unusual Evolution of a Frequent Complication,” Endocrinology, diabetes & metabolism case reports 2018 (2018): 18–0065, 10.1530/EDM-18-0065. PMC6075372 30083351

[27] S. B. Miller , B. A. Brandes , R. R. Mahmarian , and J. R. Durham , “Verrucous Carcinoma of the Foot: A Review and Report of Two Cases,” Journal of Foot and Ankle Surgery 40, no. 4 (2001): 225–231, 10.1016/s1067-2516(01)80022-3. 11924683

[28] Y. Nakamura , K. Kashiwagi , A. Nakamura , and M. Muto , “Verrucous Carcinoma of the Foot Diagnosed Using p53 and Ki‐67 Immunostaining in a Patient With Diabetic Neuropathy,” American Journal of Dermatopathology 37, no. 3 (2015): 257–259, 10.1097/dad.0000000000000117. 24901474

[29] L. C. Nwabudike and D. Gutu , “Missing the Wood for the Trees: A Case of Recalcitrant Foot Ulcer,” Clinical and Experimental Dermatology 46, no. 3 (2020): 562–564, 10.1111/ced.14455. 32969072

[30] S. Panda , S. Khanna , S. K. Singh , and S. K. Gupta , “Squamous Cell Carcinoma Developing in a Diabetic Foot Ulcer,” International Journal of Lower Extremity Wounds 10, no. 2 (2011): 101–103, 10.1177/1534734611412001. 21693447

[31] H. C. Park , H. I Kwon , H. W. Kim , J. E. Kim , Y. S. Ro , and J. Y. Ko , “A Digital Squamous Cell Carcinoma Mimicking a Diabetic Foot Ulcer, With Early Inguinal Metastasis and Cancer‐Related Lymphedema,” American Journal of Dermatopathology 38, no. 2 (2016): e18–e21, 10.1097/dad.0000000000000412. 26825165

[32] S. B. Park , Y. K. Lee , D. H. Lee , S. M. Kim , H. W. Bae , and Y. Uk Park , “Early Transformed Diabetic Foot Ulcer Into a Malignancy: A Case Report,” Journal of Korean Foot and Ankle Society 22, no. 2 (2018): 78–81, 10.14193/jkfas.2018.22.2.78.

[33] S. Pavlovic , D. Surowiec , and M. Braniecki , “Marjolin's Ulcer of the Hallux,” Journal of the American Podiatric Medical Association 103, no. 3 (2013): 250–253, 10.7547/1030250. 23697735

[34] K. E. Penera , K. A. Manji , A. B. Craig , R. A. Grootegoed , T. R. Leaming , and G. A. Wirth , “Atypical Presentation of Verrucous Carcinoma: A Case Study and Review of the Literature,” Foot & Ankle Specialist 6, no. 4 (2013): 318–322, 10.1177/1938640013489341. 23687342

[35] A. Scatena , V. Zampa , G. Fanelli , E. Iacopi , and A. Piaggesi , “A Metastatic Squamous Cell Carcinoma in a Diabetic Foot: Case Report,” International Journal of Lower Extremity Wounds 15, no. 2 (2016): 155–157, 10.1177/1534734616640358. 27048180

[36] N. Storer , “Sarcomatoid Squamous Cell Carcinoma With Overlying Bowen’s Disease in a Patient Presenting With Chronic Toe Ulceration in Diabetes,” Diabetic Foot 25, no. 3 (2022): 18–21.

[37] J. Tisch Mendes and R. J. Klein , “The Value of a Biopsy in Chronic Ulcers: A Case Series,” Wounds 34, no. 4 (2022): 119–123, 10.25270/wnds/2022.119123. 35452409

[38] D. H. Truong and E. M. Nasser , “Nonhealing Wounds With Verrucous Presentation in the Neuropathic Patient: A Report of Two Cases,” Journal of the American Podiatric Medical Association 109, no. 6 (2019): 471–476, 10.7547/17-153. 31755775

[39] J. P. Van Geertruyden , C. Olemans , M. Laporte , and J.‐C. Noël , “Verrucous Carcinoma of the Nail Bed,” Foot & Ankle International 19, no. 5 (1998): 327–328, 10.1177/107110079801900511. 9622425

[40] T. C. Vlahovic , T. L. Klimaz , M. K. Piemontese , and K. M. Zinszer , “Plantar Verrucous Carcinoma: An Unusual Case of Bone Invasion and Osteomyelitis,” Advances in Skin & Wound Care 22, no. 12 (2009): 554–556, 10.1097/01.Asw.0000363475.33364.20. 19935131

[41] E. Zielonka , D. Goldschmidt , and S. de Fontaine , “Verrucous Carcinoma or Epithelioma Cuniculatum Plantare,” European Journal of Surgical Oncology 23, no. 1 (1997): 86–87, 10.1016/s0748-7983(97)80151-5. 9066754

[42] P. L. Chalya , J. B. Mabula , P. Rambau , et al., “Marjolin's Ulcers at a University Teaching Hospital in Northwestern Tanzania: A Retrospective Review of 56 Cases,” World Journal of Surgical Oncology 10, no. 1 (2012): 38, 10.1186/1477-7819-10-38. PMC3292918 22336561

[43] M. D. Fleming , J. L. Hunt , G. F. Purdue , and J. Sandstad , “Marjolin's Ulcer: A Review and Reevaluation of a Difficult Problem,” Journal of Burn Care & Rehabilitation 11, no. 5 (1990): 460–469, 10.1097/00004630-199009000-00016. 2246317

[44] I. G. Knackfuss , V. Giordano , A. L. Godoy‐Santos , N. C. Fernandes , and O. P Camargo , “Squamous Cell Carcinoma in the Foot: Case Series and Literature Review,” Acta Ortopédica Brasileira 26, no. 2 (2018): 108–111, 10.1590/1413-785220182602187183. PMC5962287 29844733

[45] J. F. Grady , M. G. Garoufalis , C. J. Moore , and R. Nasadowski , “Squamous Cell Carcinoma: A Pedal Case Presentation,” Journal of Foot Surgery 29, no. 4 (1990): 375–378. 2229915

[46] I. R. Bristow , M. L. Reynolds , and M. Young , “Malignant Melanoma Misdiagnosis in the Foot. What Can We Learn From the Literature?,” Journal of Foot and Ankle Research 19, no. 2 (2026): e70162, 10.1002/jfa2.70162. PMC13239080 42165691

[47] G. Akkus and M. Sert , “Diabetic Foot Ulcers: A Devastating Complication of Diabetes Mellitus Continues Non‐Stop in Spite of New Medical Treatment Modalities,” World Journal of Diabetes 13, no. 12 (2022): 1106–1121, 10.4239/wjd.v13.i12.1106. PMC9791571 36578865

[48] K. Khan , C. Schafer , and J. Wood , “Marjolin Ulcer: A Comprehensive Review,” Advances in Skin & Wound Care 33, no. 12 (2020): 629–634, 10.1097/01.Asw.0000720252.15291.18. 33208661

[49] B. Pekarek , S. Buck , and L. Osher , “A Comprehensive Review on Marjolin's Ulcers: Diagnosis and Treatment,” Journal of the American College of Certified Wound Specialists 3, no. 3 (2011): 60–64, 10.1016/j.jcws.2012.04.001. PMC3601857 24525526

[50] E. Copcu , A. Aktas , N. Şişman , and Y. Oztan , “Thirty‐One Cases of Marjolin's Ulcer,” Clinical and Experimental Dermatology 28, no. 2 (2003): 138–141, 10.1046/j.1365-2230.2003.01210.x. 12653697

[51] B. Baldursson , B. Sigurgeirsson , and B. Lindelöf , “Venous Leg Ulcers and Squamous Cell Carcinoma a Large‐Scale Epidemiological Study,” British Journal of Dermatology 133, no. 4 (1995): 571–574, 10.1111/j.1365-2133.1995.tb02707.x. 7577586

[52] P. Pit'hová , “Differences in Ulcer Location in Diabetic Foot Syndrome,” Vnitrní Lékarství 53, no. 12 (2007): 1278–1285. 18357863

[53] B. Aksoy and H. M. Aksoy , “Marjolin Ulcer: A Rare Clinical Entity That Every Health Professional Should Be Informed About: A Narrative Review,” Discover Oncology 16, no. 1 (2025): 1109, 10.1007/s12672-025-02948-8. PMC12167728 40517333

[54] O. Ogunlana , V. K. Panchbhavi , W. B. Norbury , and M. Raji , “Squamous Cell Carcinoma of the Foot Occurring in a Wart,” Foot & Ankle Specialist 16, no. 1 (2023): 57–59, 10.1177/19386400211070431. 35016565

[55] R. Moraga , T. Hopkins , G. S. Sclamberg , and E. S. Gallo , “Exploring the Oncogenic Potential of Human Papillomavirus in Subungual and Plantar Squamous Cell Carcinoma: A Review of the Literature,” Cancers (Basel) 17, no. 18 (2025): 3029, 10.3390/cancers17183029. PMC12468373 41008871

[56] S. A. Ederaine , J. L. Dominguez , J. A. Harvey , et al., “Survival and Glycemic Control in Patients With Co‐Existing Squamous Cell Carcinoma and Diabetes Mellitus,” Future Science OA 7, no. 5 (2021): FSO683, 10.2144/fsoa-2020-0150. PMC8147738 34046188

[57] S. T. Chen , T. Li , J. Han , A. A. Qureshi , and E. Cho , “Type 2 Diabetes Mellitus and Risk of Cutaneous Squamous Cell Carcinoma,” Journal of the American Academy of Dermatology 75, no. 4 (2016): 831–834, 10.1016/j.jaad.2016.05.013. PMC5074689 27646740

[58] R. Pop‐Busui , ADA Clinical Compendia Series, in Diagnosis and Treatment of Painful Diabetic Peripheral Neuropathy, (American Diabetes Association 2022). 35544662

