# Supplementary material for: Squamous Cell Carcinoma in the Diabetic Foot: A Review of Published Case Reports
Source: J Foot Ankle Res. 2026 Oct 26;19(4):e70217. doi: 10.1002/jfa2.70217 (PMC13704852; doi:10.1002/jfa2.70217)
Supplement: Supplementary file 1 [file JFA2-19-e70217-s001.docx]

**Supplementary File 3** Assessment of methodological quality

The methodological quality of the 31 included case reports was generally high, with a mean JBI score of 7.2/8, a median of 7/8, and a range of 5–8. Overall, 13 studies (42%) were rated as high quality (8/8), 11 (35%) as good quality (7/8), and 7 (23%) as moderate quality (≤6/8), with no studies classified as low quality. Most reports provided detailed descriptions of patient demographics, clinical presentation, and diagnostic processes, with histopathological confirmation consistently reported. Reporting of interventions was similarly strong, with most studies describing surgical management and associated outcomes. Key strengths across the dataset included comprehensive clinical characterisation and clear take-home messages, particularly highlighting the importance of biopsy in non-healing or atypical ulcers. However, several limitations were identified. Reporting of adverse events was inconsistent, and long-term follow-up data were frequently limited or absent. Variability in timeline reporting and completeness of post-intervention outcomes was also evident, particularly in older case series and studies involving diagnostic ambiguity or benign mimics. Despite these limitations, no studies were excluded on methodological grounds, supporting the overall robustness of the evidence base while highlighting the need for more consistent reporting of longitudinal outcomes in future case report research.

**Data extraction**

Data extraction support was assisted by Microsoft Copilot (Microsoft Corporation). Copilot was used to facilitate structured information extraction and to assist in populating the Joanna Briggs Institute (JBI) critical appraisal checklist for each included case report. AI-assisted outputs were reviewed, verified, and amended where necessary by the reviewer to ensure accuracy and consistency with the source publications. Final appraisal decisions and scoring were made exclusively by the authors.

| **Study** | **Demographics** | **History / timeline** | **Clinical condition** | **Diagnostic tests** | **Intervention / treatment** | **Post-intervention condition** | **Adverse / unanticipated events** | **Takeaway lessons** | **Score / 8** |
| --- | --- | --- | --- | --- | --- | --- | --- | --- | --- |
| **Ahmedy 2019** | Y | Y | Y | Y | Y | Y | Y | Y | **8/8** |
| **Bentley 2013** | Y | Y | Y | Y | Y | Y | U | Y | **7/8** |
| **Bhushan 2001** | Y | Y | Y | Y | Y | Y | U | Y | **7/8** |
| **Cavaliere 2018** | Y | Y | Y | Y | Y | Y | Y | Y | **8/8** |
| **Chiao 2014** | Y | Y | Y | Y | Y | Y | U | Y | **7/8** |
| **Chong 2001** | Y | Y | Y | Y | U | U | U | Y | **5/8** |
| **Christopher 2004** | Y | Y | Y | Y | Y | Y | U | Y | **7/8** |
| **Di Palma 2018** | Y | Y | Y | Y | Y | Y | U | Y | **7/8** |
| **Ho 2000** | Y | Y | Y | Y | Y | Y | U | Y | **7/8** |
| **Holgado 2000** | Y | Y | Y | Y | Y | Y | U | Y | **7/8** |
| **Jones 2020** | Y | Y | Y | Y | Y | Y | U | Y | **7/8** |
| **Klein 2022 (Case series)** | Y | Y | Y | Y | Y | Y | U | Y | **7/8*** |
| **Kong 2008** | Y | Y | Y | Y | Y | Y | U | Y | **7/8** |
| **Larijani 2017** | Y | Y | Y | Y | Y | Y | U | Y | **7/8** |
| **Mantovani 2018** | Y | Y | Y | Y | Y | Y | U | Y | **7/8** |
| **McIntyre 2008** | Y | Y | Y | Y | Y | Y | U | Y | **7/8** |
| **Miller 2001** | Y | Y | Y | Y | Y | Y | U | Y | **7/8*** |
| **Nakamura 2015** | Y | Y | Y | Y | Y | Y | U | Y | **7/8** |
| **Nwabudike 2021** | Y | Y | Y | Y | U | U | U | Y | **5/8** |
| **Panda 2011** | Y | Y | Y | Y | Y | Y | U | Y | **7/8** |
| **Park HC 2016** | Y | Y | Y | Y | Y | U | Y | Y | **7/8** |
| **Park SB 2018** | Y | Y | Y | Y | Y | Y | Y | Y | **8/8** |
| **Pavlovic 2013** | Y | Y | Y | Y | Y | Y | Y | Y | **8/8** |
| **Penera 2013** | Y | Y | Y | Y | Y | Y | U | Y | **7/8** |
| **Russo 2022** | Y | Y | Y | Y | Y | Y | U | Y | **7/8** |
| **Scatena 2016** | Y | Y | Y | Y | U | Y | Y | Y | **7/8** |
| **Storer 2022** | Y | Y | Y | Y | Y | Y | U | Y | **7/8** |
| **Truong 2019** | Y | Y | Y | Y | Y | Y | U | Y | **7/8*** |
| **van Geertruyden 1998** | Y | Y | Y | Y | Y | Y | U | Y | **7/8** |
| **Vlahovic 2009** | Y | Y | Y | Y | Y | Y | U | Y | **7/8** |
| **Zielonka 1997** | Y | Y | Y | Y | Y | Y | U | Y | **7/8** |

Y = Yes (criterion met); N = No (criterion not met); U = Unclear (insufficient detail to determine).
