# Supplementary material for: Squamous Cell Carcinoma in the Diabetic Foot: A Review of Published Case Reports
Source: J Foot Ankle Res. 2026 Oct 26;19(4):e70217. doi: 10.1002/jfa2.70217 (PMC13704852; doi:10.1002/jfa2.70217)
Supplement: Supplementary file 3 [file JFA2-19-e70217-s003.docx]

**Supplementary File 2 - Search strategy**

**Search Terms [Medline, CINAHL, SPORTDiscus]**

| **a** | 1 | Subject term | exp. Squamous cell carcinoma |
| --- | --- | --- | --- |
|  | 2 | Subject term | exp. Carcinoma Verrucous |
|  | 3 | Keyword | “Carcinoma cuniculatum” **or** “Papillary carcinoma” **or** “Epidermoid carcinoma” **or** “Marjolin* ulcer” |
|  |  |  |  |
| **b** | 4 | Subject | exp. Diabetes mellitus |
|  | 5 |  | exp. Diabetic foot |
|  | 6 | Subject | exp. Foot ulcer |
|  | 7 | Subject | exp. Leg ulcer |
|  | 8 | Keyword | ”diab* foot ulcer” **or** “neuro* ulcer” **or** “ischemic ulcer” **or** “venous stasis ulcer” **or** “pressure ulcer” |
| **c** | 9 | Keyword | “Lower extremi*” **or** “Lower limb*” **or** Foot |
| **d** | 10 | Combine | 1 **or** 2 **or** 3 |
|  | 11 | Combine | 4 **or** 5 **or** 6 **or** 7 **or** 8 **or** 9 |
|  | 12 | Combine | 10 **and** 11 **and** 12 |

**Scopus search terms**

| **a** | 1 | Keyword | "Squamous cell carcinoma" **or** "Carcinoma Verrucous" **or** "Carcinoma cuniculatum" **or** "Papillary carcinoma" **or** "Epidermoid carcinoma" **or** "Marjolin* ulcer" |
| --- | --- | --- | --- |
|  | 2 | Keyword | diabet* **or** "diab* foot ulcer" **or** "neuro* ulcer" **or** "ischemic ulcer" **or** "venous stasis ulcer" **or** "pressure ulcer" **or** "foot ulcer" **or** "leg ulcer" |
|  | 3 | Keyword | "Lower extremi*" **or** "Lower limb*" **or** foot |
| **b** | 4 | Combine | 1 **and** 2 **and** 3 |
